# Dysregulation of Suppressor of Cytokine Signaling 3 in Keratinocytes Causes Skin Inflammation Mediated by Interleukin-20 Receptor-Related Cytokines

**DOI:** 10.1371/journal.pone.0040343

**Published:** 2012-07-05

**Authors:** Ayako Uto-Konomi, Kosuke Miyauchi, Naoko Ozaki, Yasutaka Motomura, Yoshie Suzuki, Akihiko Yoshimura, Shinobu Suzuki, Daniel Cua, Masato Kubo

**Affiliations:** 1 Laboratory for Signal Network, Research Center for Allergy and Immunology, RIKEN Yokohama Institute, Yokohama, Kanagawa, Japan; 2 Department of Molecular and Cellular Biology, Kobe Pharma Research Institute, Nippon Boehringer Ingelheim Co., Ltd., Kobe, Hyogo, Japan; 3 Department of Microbiology and Immunology, Keio University School of Medicine, Tokyo, Japan; 4 Schering-Plough Biopharma, Palo Alto, California, United States; 5 Division of Molecular Pathology, Research Institute for Biological Science, Tokyo University of Science, Noda, Chiba, Japan; Instituto de Biofisica Carlos Chagas Filho, Universidade Federal do Rio de Janeiro, Brazil

## Abstract

Homeostatic regulation of epidermal keratinocytes is controlled by the local cytokine milieu. However, a role for suppressor of cytokine signaling (SOCS), a negative feedback regulator of cytokine networks, in skin homeostasis remains unclear. Keratinocyte specific deletion of *Socs3* (*Socs3* cKO) caused severe skin inflammation with hyper-production of IgE, epidermal hyperplasia, and S100A8/9 expression, although *Socs1* deletion caused no inflammation. The inflamed skin showed constitutive STAT3 activation and up-regulation of IL-6 and IL-20 receptor (IL-20R) related cytokines, IL-19, IL-20 and IL-24. Disease development was rescued by deletion of the *Il6* gene, but not by the deletion of *Il23*, *Il4r,* or *Rag1* genes. The expression of IL-6 in *Socs3* cKO keratinocytes increased expression of IL-20R-related cytokines that further facilitated STAT3 hyperactivation, epidermal hyperplasia and neutrophilia. These results demonstrate that skin homeostasis is strictly regulated by the IL-6-STAT3-SOCS3 axis. Moreover, the SOCS3-mediated negative feedback loop in keratinocytes has a critical mechanistic role in the prevention of skin inflammation caused by hyperactivation of STAT3.

## Introduction

The suppressor of cytokine signaling (SOCS) family of proteins plays a role in the negative regulation of cytokine-JAK-STAT signaling by inhibiting JAK tyrosine kinase activity. There are eight proteins in this family, each of which has a central SH2 domain and a C-terminal 40-amino-acid conserved domain called the SOCS box. SOCS1 inhibits STAT1 activation in the IFN-γ signaling cascade, while SOCS3 is a major negative regulator of IL-6-STAT3 signaling [1], [2]. Additionally, SOCS3 negatively regulates skin wound healing through inhibition of the gp130-STAT3 pathway [3], suggesting a pivotal role for SOCS proteins in skin inflammatory responses. However, the regulatory role of SOCSs in the maintenance of skin homeostasis remains unclear.

The skin consists of two major layers, the epidermis and the dermis. The epidermis consists of keratinocytes, which proliferate in the basal layer and differentiate into cells that migrate towards the outer layer to form the stratified epithelium that provides the skin barrier. The epidermis is in a continuous equilibrium of growth and differentiation and has the remarkable capacity for complete self-renewal, relying on its rich reservoir of stem cells. The dermis contains mainly fibroblasts and a large population of immune cells, along with structures important for skin function including blood vessels, nerves, hair follicles, and glands. The dermis also provides the epidermis with mechanical support and nutrients [4],[5]. Epidermal homeostasis is dependent on proper repair after injury and on maintenance of a tight junction with the underlying basement membrane, both of which are precisely regulated by various cytokines. Keratinocytes are now proposed to play important roles in the regulation of skin homeostasis by producing a variety of cytokines [5]. TNF-α, IL-1 and IL-6 have been shown to have regulatory roles in skin wound healing, and also in skin permeability [6], [7], [8]. For example, IL-6 deficiency caused delayed skin repair, and topical application of IL-6 to barrier-disrupted skin enhanced repair of the skin barrier [8].

STAT3 is one of the key components for IL-6 receptor signaling, and it is thought to be an important transcription factor in skin homeostasis [8], [9]. Activated (phosphorylated) STAT3 expression is enhanced at sites of injured epidermis compared with the normal epidermis, and STAT3 related signaling also affects the survival of keratinocyte stem cells, which are important for keratinocyte renewal and wound healing. Besides IL-6, other cytokines/growth factors also stimulate the phosphorylation of STAT3 and the subsequent STAT3 mediated nuclear signaling. Thus a sophisticated cytokine network is required for the homeostatic events in the proliferation and differentiation of keratinocytes. Disruption of this homeostasis has pathological consequences. For example, the overexpression of active-*Stat3* resulted in impaired wound healing and increased keloid pathogenesis [9] and augmented the development of spontaneous psoriatic skin disease [10]. Therefore, dysregulation of STAT3 activation results in the breakdown of keratinocyte homeostasis, sometimes leading the development of skin carcinogenesis [11].

Phosphorylation of STAT3 is regulated by IL-6, IL-10, EGF and many other cytokines, including the interleukin-20 receptor (IL-20R) related cytokines, IL-19, IL-20, and as well as IL-24 [12]. The IL-20RI is composed of two chains, IL-20RA and IL-20RB, and its ligands are IL-19, IL-20 and IL-24, which are highly expressed in keratinocytes [13]. These cytokines are important in the manifestation of psoriatic lesions [14] and, recently, an association of polymorphisms of IL-20 with psoriasis has been described [15]. IL-19, IL-20 and IL-24 are reported to induce epidermal hyperplasia and STAT3 activation in the reconstituted human epidermis [16], and are highly expressed in psoriatic inflammatory sites [17].

In the present study, we investigated the role of SOCS3 in keratinocyte function using keratinocyte-specific SOCS3 gene deficient mice, and found that these mice spontaneously developed a severe form of skin inflammation. Here we identified a critical role for SOCS3 as a negative regulator of STAT3 hyperactivation in keratinocytes that restored skin homeostasis. Moreover, in the absence of SOCS3, excessive IL-6 signaling resulted in skin lesions that were also strongly correlated with increased IL-19/IL-24 cytokines and the expression levels of anti-microbial peptides, β-defensin and S100A8/A9.

## Results

### Loss of SOCS3 in keratinocytes resulted in skin inflammation

**Figure 1:**
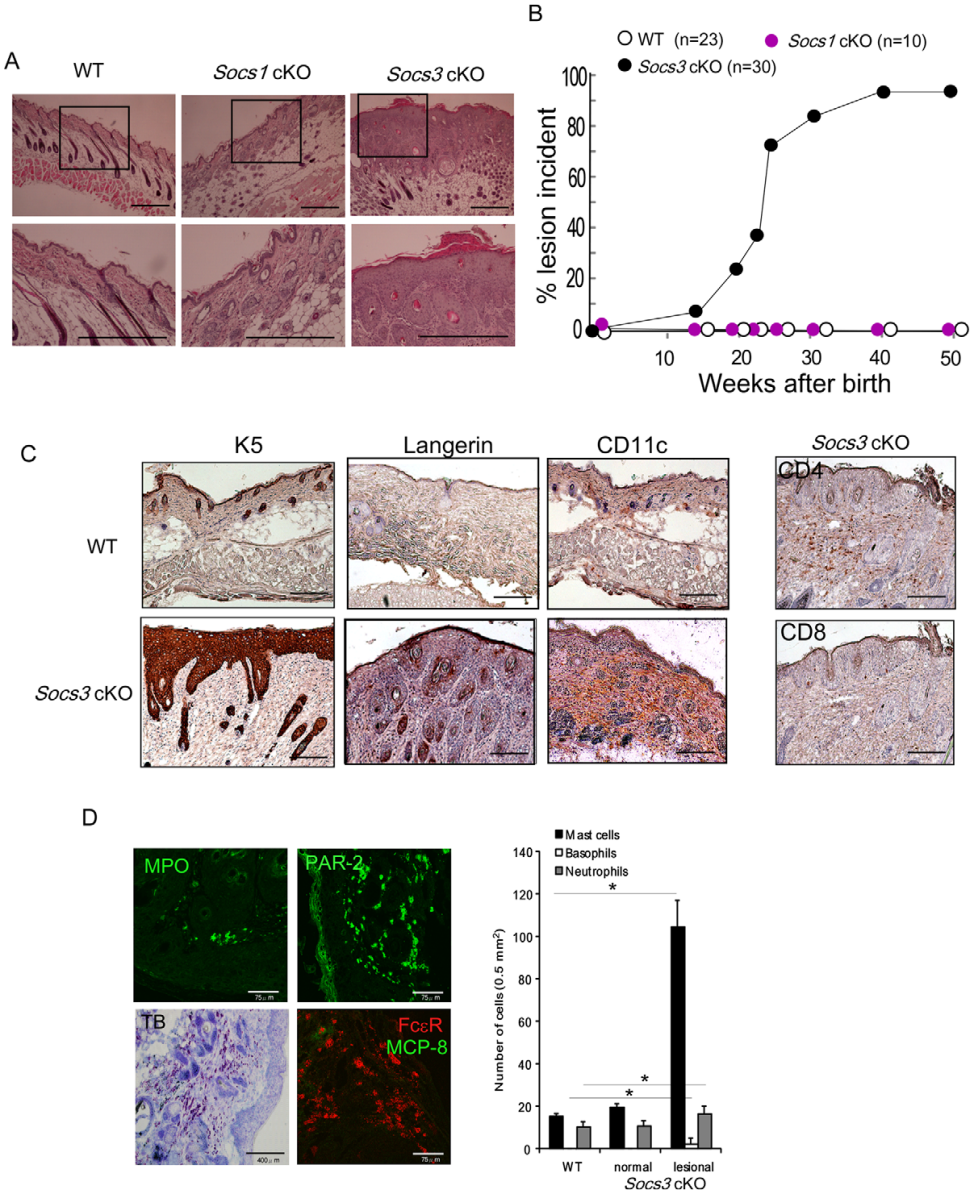
Socs3 cKO spontaneously developed epidermal hyperplasia and skin inflammation. A) Upper and bottom panels show histopathological analysis of K5-Cre (WT) (left), *Socs1* cKO (center) and *Socs3* cKO (right) mice skin with hematoxylin and eosin staining. Magnification of upper and lower panels are x40 and x100, respectively. Scale bar in each section indicates 750 υm. B) Percentage of mice showing skin lesion incidences in wild type (WT) (open circle), *Socs1* cKO (closed purple circle) and *Socs3* cKO (closed-black circle) mice. Disease incidence was monitored weekly up to 50 weeks after birth. Percentage of mice showing incidence of disease is shown on the Y axis at each time point. The number of mice used in each group is indicated in the figure. C) Localization of keratinocytes (K5), Langerhans cells (Langerin), and CD11c^+^ dendritic cells (CD11c) in epidermis and dermis of the diseased *Socs3* cKO skin was assessed by immunohistlogical staining (x40) (left panel). Wild type (WT) mice skin was stained as a control. CD4 and CD8 positive T cells were also identified in epidermis and dermis of the diseased *Socs3* cKO skin (right panel). Scale bar in each section indicates 750 υm. D) Neutrophil number was confirmed by counting MPO positive cells in epidermis and dermis of the diseased *Socs3* cKO skin by immunohistlogical staining (x400) (upper left panel). PAR-2, TB staining, FceR and MCP-8 positive cells in epidermis and dermis of the diseased *Socs3* cKO skin are shown (upper right, lower left, and lower right panel, respectively). Scale bar in each section indicates 75 υm. Number of neutrophils, mast cells and basophils in epidermis and dermis are shown in the right bar graphs. *P<0.01. Data are the mean from three independent experiments. Error bars are SD.

To examine the role of SOCS proteins in the epidermis, we generated keratin 5-specific *Socs*1 and *Socs3* conditional knockout mice (*Socs*1 cKO and *Socs3* cKO respectively; See Materials and Methods). At age 40 weeks or older, mice lacking SOCS3 in keratinocytes spontaneously developed a severe form of skin inflammation, with a disease incidence of >90% (**Fig.** **1A&B**). Histopathological analysis revealed epidermal hyperplasia and massive leukocyte infiltration in the skin of the *Socs3* cKO mice, whereas the deletion of *Socs1* did not cause such inflammation. These findings suggested that SOCS3 expression in keratinocytes is required for the maintenance of normal skin. Additionally, we analyzed the cell types in the skin lesions (**Fig.** **1C&D**). Keratin 5 (K5) staining showed a thickened epithelium in *Socs3* cKO mice as a result of an extensive expansion of keratinocytes. Compared to the control K5-Cre mice (with a wild type *Socs3* locus), *Socs3* cKO mice displayed an increased infiltration of Langerin^+^ Langerhans cells and CD11c^+^ dendritic cells (DCs). We also identified an increased number of CD4^+^ but not CD8^+^ T cells in the dermis of *Socs3* cKO mice (**Fig.** **1C, right panel**). In addition, we performed an immunohistochemical staining of neutrophils, connective tissue type mast cells and basophils in the epidermis and dermis of *Socs3* cKO mice, using toluidine blue or antibodies for myeloperoxidase (MPO), PAR-2, FcεR and MCP-8 (**Fig.** **1D**). Compared to the wild type littermates, elevated numbers of mast cells were identified in both the epidermis and dermis of *Socs3* cKO mice, and there was also an increased infiltration of neutrophils and basophils. These findings indicated that the loss of SOCS3 rendered the mice prone to spontaneous skin inflammation.

### The skin inflammation in Socs3 cKO mice is T and B cell independent

**Figure 2:**
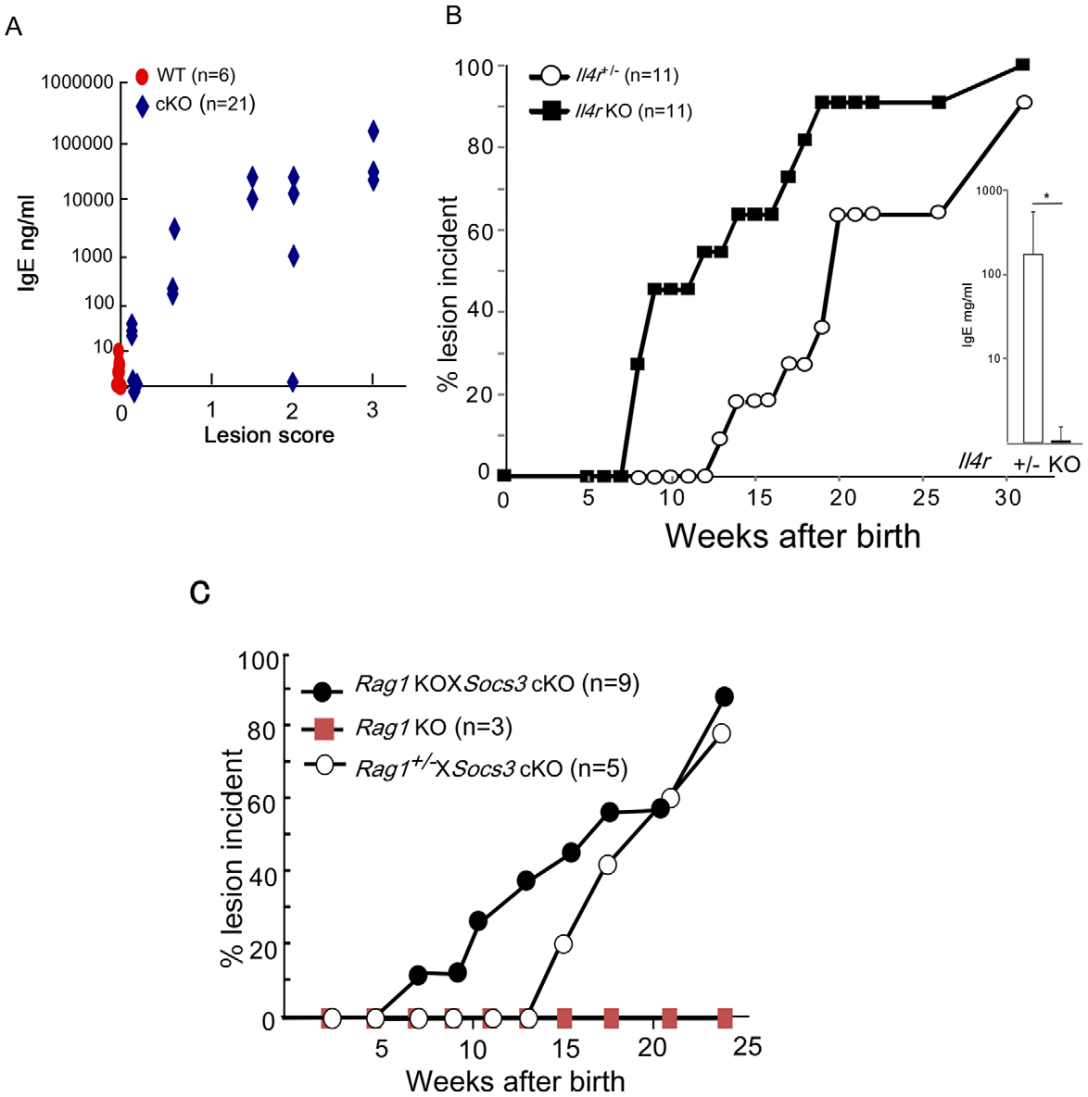
T cell responses are not required for initiation of the skin disease in Socs3cKOmice. A) Serum level of IgE was measured in wild type (WT) and *Socs3* cKO mice and is shown on the Y axis. X axis indicates skin disease severity (Lesion score). Red symbols indicate WT mice (n = 6) and blue symbols indicate *Socs3* cKO with or without skin lesions (n = 21). B) Lesion incidence was examined in *Socs3* cKO with *Il4r* ^+/−^ (shown as *Il4r* ^+/−^ in the figure, open circle, n = 11) and *Il4r* null *Socs3* cKO (shown as *Il4* KO in the figure, closed circle, n = 11). Serum IgE in individual *Socs3* cKO with *Il4r^+/^* ^−^ (+/−) and *Socs3* cKO with *Il4r* KO mice (KO) was measured at 30 weeks of age and the mean +/− SD is shown (right). C) Role of T-B cells in the development of the skin lesions in *Socs3* cKO mice. Lesion incidence was examined in *Rag1* KO (red-square, n = 3), *Socs3* cKO with *Rag1* KO (Rag1 KOX*Socs3* cKO, closed circles, n = 9), and *Socs3* cKO with *Rag*1^+/−^ (*Rag1* ^+/−^X*Socs3* cKO, open circles, n = 5) mice. Each mouse strain was monitored until 24 weeks after birth (X axis). The percentage of mice with lesions is shown on the Y axis at each time point after birth.

We examined whether this skin inflammation was due to an atopic type Th2 response and, interestingly, we found that total serum levels of the prototypical Th2-type immunoglobulin, IgE, were markedly increased in the diseased *Socs3* cKO mice, but not in pre-disease *Socs3* cKO mice. Moreover, we observed that there was a positive correlation between the IgE levels and the severity of inflammation in the diseased *Socs3* cKO mice (**Fig.** **2A**). Therefore, in order to understand if any atopic response associates with this skin pathology, the *Socs3* cKO mice were crossed with *Il4 receptor (Il4r)* KO mice and monitored for the spontaneous development of skin inflammation over time. We found that *Il4r* KO mice still developed skin lesions similar to those observed in *Il4r* ^+/−^ mice, even with complete attenuation of the increased serum IgE levels (**Fig.** **2B**). This further demonstrates that the skin inflammation observed in *Socs3* cKO mice completely segregated from the IgE-mediated as well as the IL-4/IL-13-mediated responses.

As shown in Fig. 1D, we found massive infiltration of CD4^+^ cells but not CD8^+^ T cells in the dermis of the diseased skin. We therefore asked whether T cells were involved in the actual development of skin lesions, or were only needed for maintenance of the disease situation in an ongoing inflammation. To answer the question, we crossed *Socs3* cKO mice onto a *Rag1* KO background, in which T and B cells were completely absent. Interestingly, spontaneous skin lesions still occurred in these mice (**Fig.** **2C**). Our results indicate that the development of skin lesion in *Socs3* cKO mice was independent of CD4 T cells, and that CD4 T cells in the lesions might accumulate locally as a result of the inflammation.

### Cytokine profile in the lesional skin of Socs3 cKO mice

**Figure 3:**
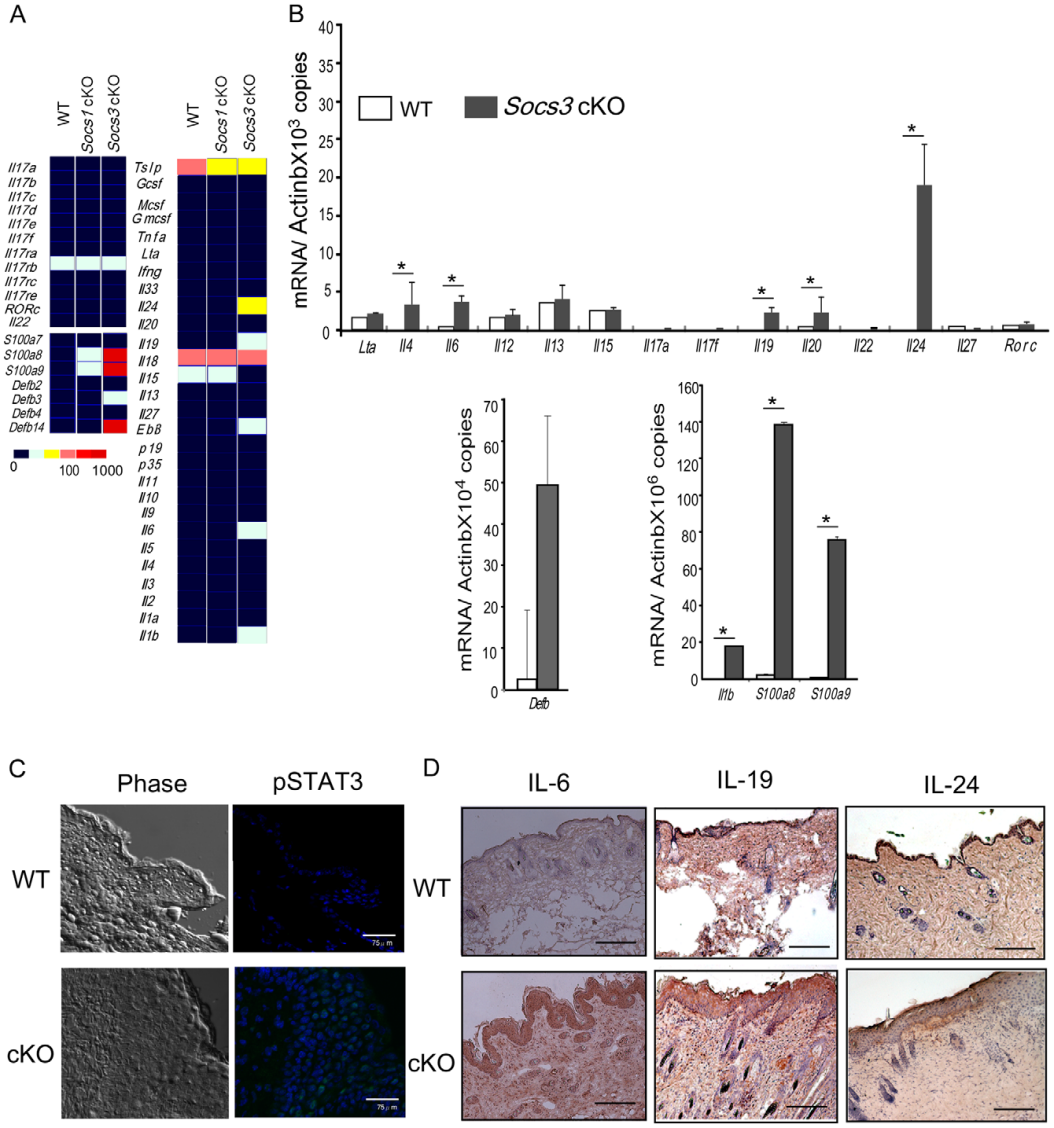
Cytokine expression in the diseased skin. A) Expression profiles. RNA was prepared from skin of K5-Cre (WT), *Socs1* cKO, and the diseased *Socs3* cKO mice, and analyzed using a TAQMAN™ real-time quantitative PCR system. Copy numbers are depicted by the color indicators shown on the lower left. B) Quantitative RT-PCR analysis of the cytokine and *Rorc* panel (upper), *Defb* (lower left) and *S100a8* and *S100a9* (lower right) expression. Skin from K5-Cre (WT, open column) and *Socs3* cKO (closed column) mice was analyzed by SYBR green real-time qPCR. Data are normalized to β-actin mRNA copy number and the mean and SEM (n = 5) are indicated. Statistical significance was determined using the Student's t-test. * p<0.05. C) Skin sections from K5-Cre control (WT) and the diseased *Socs3* cKO mice (cKO) were stained with Alexa 488 labeled anti-pSTAT3. Left panels represent phase contrast of skin section, and right panels represent pSTAT3 (green) in the skin section (x400). Scale bar in each section indicates 75 υm. D) Protein expression of IL-6, IL-19, and IL-24 in the frozen skin sections from K5-Cre (WT) and the diseased *Socs3* cKO mice were analyzed by immunohistochemical staining (x40). Scale bar in each section indicates 750 υm.

To understand the mechanism underlying the development of skin inflammation in *Socs3* cKO mice, we examined the cytokine profiles. Cytokines, cytokine receptors, and anti-microbial peptide gene panels, that are known to play a role in skin homeostasis or diseases, were analyzed by quantitative real-time PCR in normal skin from controls and *Socs1* cKO mice, as well as in the inflamed skin of *Socs3* cKO mice. We found clear induction of IL-1β, IL-4, IL-6, IL-19, IL-20 and IL-24 expression in *Socs3* cKO derived skin (**Fig.** **3A&B**). It has been reported that the cytokines IL-19, IL-20 and IL-24 are highly expressed in the inflammatory sites of psoriasis, and may thus mediate the progression of psoriasis [13], [17]. Only STAT3, but no other STAT family member (STAT1, STAT5, or STAT6), is highly phosphorylated in the skin of *Socs3* cKO mice (**Fig.** **3C**, **Fig.** **S1**). Immunohistochemical analysis revealed that IL-6, IL-19 and IL-24 protein levels are also elevated in *Socs3* cKO mouse skin (**Fig.** **3D**). However, the inflamed skin had no expression of IL-23, IL-17A, IL-17F, IL-22, and RORγt (**Fig.** **3A&B** **Fig.** **S2**), which are known to be expressed by Th17 cells, a critical helper T cell population in various inflammatory conditions including psoriasis [18]. On the other hand, the inflamed skin expressed significantly high levels of the anti-microbial peptides; S100A8, S100A9, and β-defensin (**Fig.** **3A&B**)

**Figure 4:**
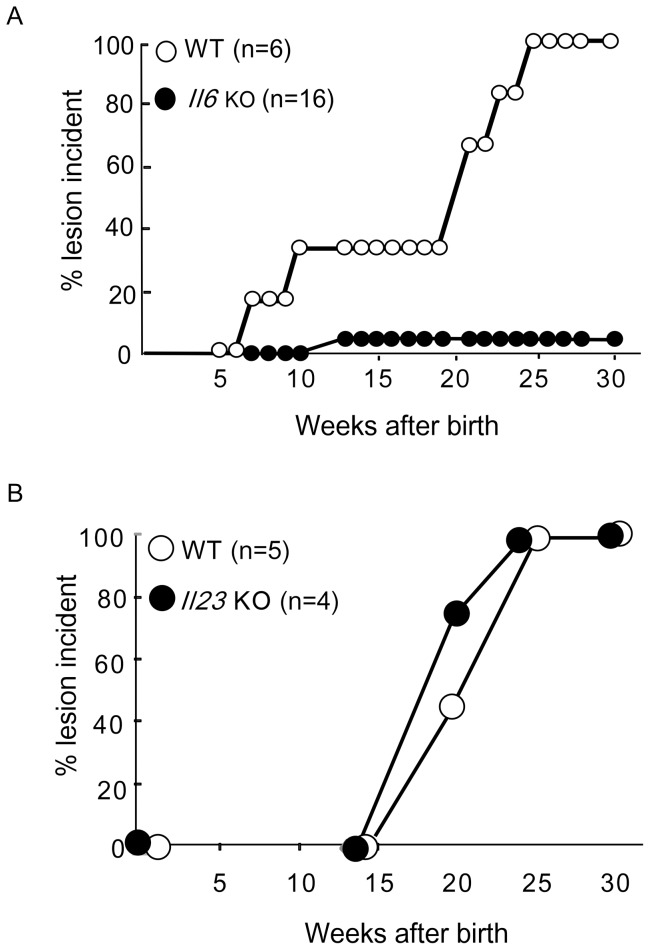
Lesion incidences in Socs3 cKO mice and in combined Il6^−/−^- or Il23^−/−^-Socs3 cKO mice. A) Lesion incidences were examined in K5-Cre mice (WT, open circle, n = 6) and *Il6* ^−/−^ *Socs3* cKO (*Il6* KO, closed circle, n = 16) mice. Each mouse strain was monitored up to 30 weeks after birth. The percentage of the mice showing disease incidence is shown on the Y axis in all examined mice at each time point. B) Lesion incidences were examined in K5-Cre mice (WT, open circle, n = 5) and *Il23* ^−/−^ *Socs3* cKO (*Il23* KO, closed circle, n = 4) mice. Each mouse strain was monitored for up to 30 weeks after birth. The percentage of the mice showing disease incidence is shown on the Y axis in all examined mice at each time point.

IL-6 plays a regulatory role in skin wound healing through its effect on skin barrier homeostasis [8]. This indicates that IL-6 might be a key cytokine to enhance the hyperactivation of STAT3 in the skin of *Socs3* cKO mice. IL-23 is also well known to be an important STAT3 cytokine related to skin disorders such as psoriasis [19], [20], [21]. Therefore, we tested *Socs3* cKO mice crossed with either *Il6* KO or *Il23* KO mice to identify which cytokine is more important for disease development. We found that crossing *Socs3* cKO mice with *Il6* KO mice markedly restored the skin condition (**Fig.** **4A**). By contrast, skin from *Socs3* cKO mice crossed with *Il23* KO mice did not show any improvement in disease development (**Fig.** **4B**). These results demonstrate that IL-6 is a key cytokine for the regulation of homeostasis in keratinocytes, and that STAT3 activation and aberrant expression of IL-19 and IL-24 may promote development of skin inflammation.

### IL-6 induces IL-20-RI related cytokines in Socs3 KO keratinocytes

**Figure 5:**
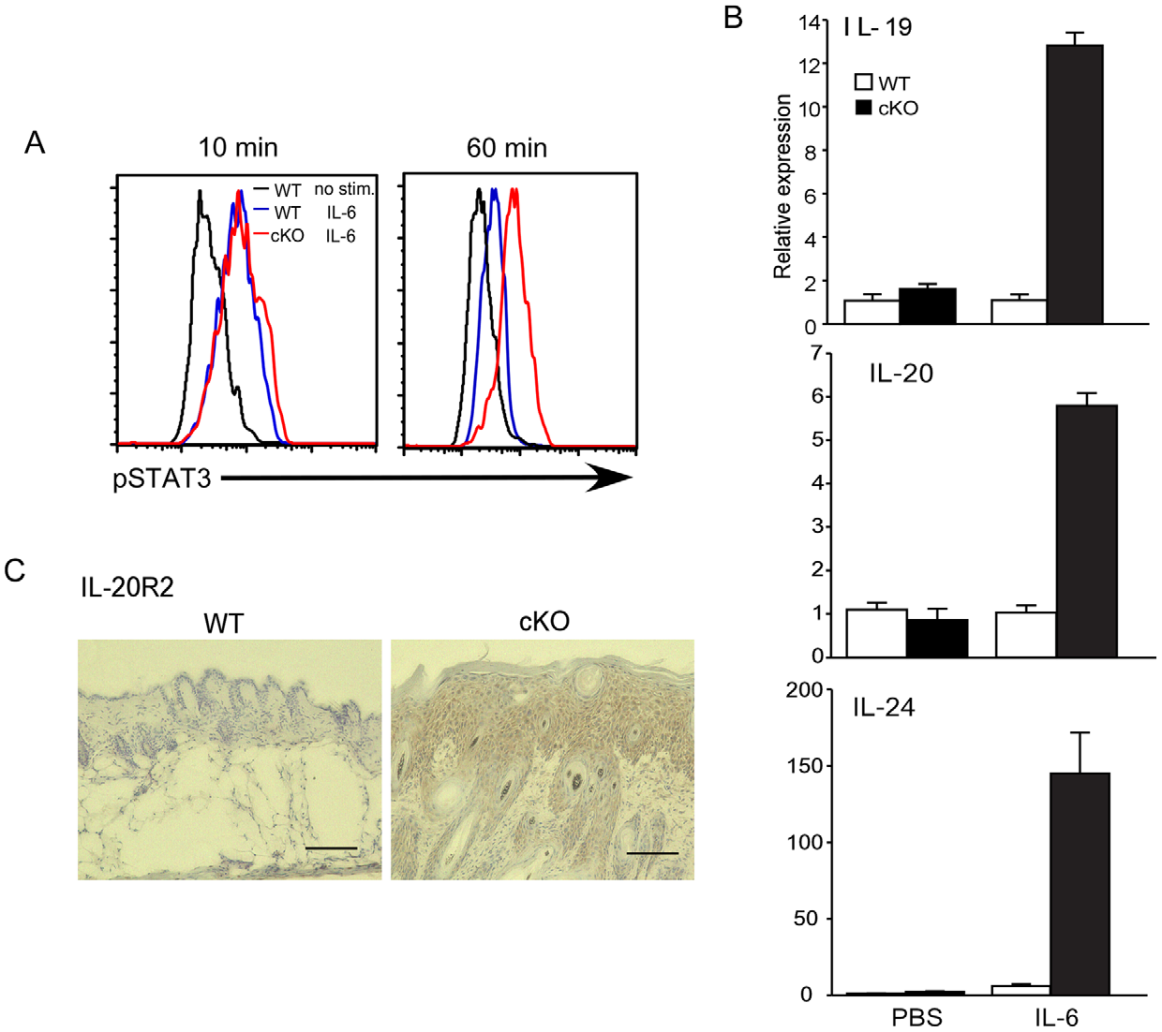
The effect of IL-6 on primary keratinocytes derived from Socs3 cKO mice. A) Mouse primary keratinocytes were stimulated with 10 ng/ml of IL-6 for 10 or 60 min and pSTAT3 was analyzed by flow cytometry. Black lines show the PBS control, blue lines show the keratinocytes from normal C57BL/6 mice, and red lines show the keratinocytes from *Socs3* cKO mice. Data are representative of 3 separate experiments. B) mRNA expression of IL-20R related cytokines in the keratinoyctes. Mouse primary keratinocytes were stimulated with or without IL-6 for 24 hrs and the expression of IL-19, IL-20 and IL-24 mRNA was investigated by quantitative RT-PCR. Open bars show the keratinocytes from C57BL/6 mice (WT), and closed bars show those of *Socs3* cKO mice. Data are relative expression to PBS-treated C57BL/6 keratinocytes. Data are mean ± SEM, n = 3. C) Increased expression of IL-20R2 protein in the diseased skin of *Socs3* cKO mice. Protein levels of IL-20R2 in the frozen skin sections from K5-Cre (WT) and the diseased *Socs3* cKO mice were analyzed by immunohistochemical staining (x200). Scale bar in each section indicates 150 υm.

As shown in Fig. 3C, the STAT3 pathway is highly active in *Socs3* deficient skin. To confirm the prolonged activation of STAT3 in the SOCS3 deficient condition, keratinocytes were isolated from wild type and pre-diseased *Socs3* cKO mice and cultured with IL-6 before analyzing their STAT3 phosphorylation status. Ten minute stimulation of KO keratinocytes induced similar levels of STAT3 phosphorylation to that of wild type keratinocytes. However, after 60 min, there was a significant reduction in STAT3 phosphorylation in control keratinocytes, whereas the phosphorylation levels in KO keratinocytes was markedly sustained (**Fig.** **5A**).

We further examined the effect of IL-6 on *Socs3* deficient keratinocytes in the induction of expression of IL-20R related cytokine genes, *Il19*, *Il20* and *Il24*. Socs3 deficient keratinocytes showed much higher IL-20RI related cytokine mRNA levels than the control keratinocytes (**Fig.** **5B**). Our results revealed a prolonged STAT3 activation in response to IL-6 in the *Socs3* deficient keratinocytes, eventually leading to the expression of IL-19, IL-20 and IL-24 by keratinocytes. Furthermore, we found enhanced expression of IL-20R2, the receptor for IL-19, IL-20, and IL-24, on the keratinocytes of the diseased Socs3 cKO mice (**Fig.** **5C**).

### IL-20R related cytokines induce epidermal hyperplasia in Socs3 cKO skin

**Figure 6:**
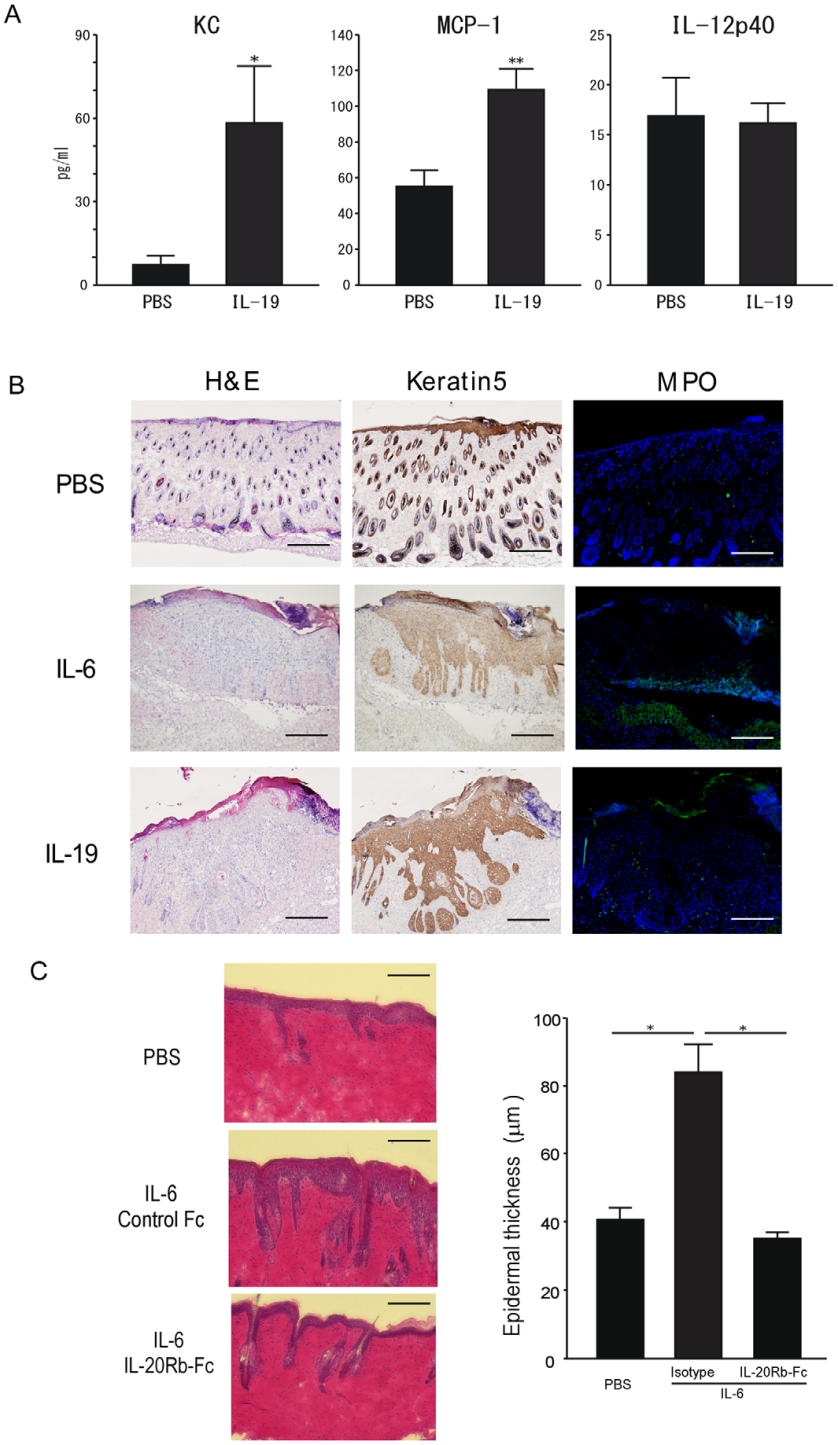
Effect of IL-19 on skin inflammation in the Socs3 cKO mice. A) Amounts of KC, MCP-1 and IL12p40 in the air pouches after IL-19 injection into C57BL/6 mice. IL-19 or PBS was injected into mouse air pouches, and chemokine concentration in the pouch lavage was measured after 5 hrs. Data are mean ± SEM, n = 5–6. B) IL-6 and IL-19 induced skin inflammation in the *Socs3* cKO mice. *Socs3* cKO mice were injected with PBS (upper rows), 10 ng of IL-6 (middle rows) or 10 ng of IL-19 (bottom rows) was injected intradermally and skin sections were obtained two weeks later. Left panels show the H&E staining, middle panels show K5 immunostaining, and right panels show MPO^+^ neutrophils (x40). Scale bar in each section indicates 750 υm. C) *Socs3* cKO mice were injected with PBS (upper rows) or 20 ng of IL-6 (middle and bottom rows) intradermally with 5 υg of control Fc or IL-20Rβ fusion Fc (IL-20Rβ-Fc). After two weeks, skin sections were stained with H&E and epidermal thickness was measured at the injection site (x200). Scale bar in each section indicates 150 υm. Bar graph (right panel) indicates the mean and SEM (n = 3) of epidermal thickness (υm).

Up-regulation of IL-20R related cytokines, IL-19, IL-20 and IL-24, has often been reported in several skin diseases [22]. We therefore examined the effect of IL-20R related cytokines on skin pathology using the air pouch system. Recombinant IL-19 was injected into the air space generated in the skin of control and *Socs3* cKO mice. Interestingly, treatment with IL-19 in the pouches of *Socs3* cKO skin induced KC and MCP-1 production, but not IL-12p40 production (**Fig.** **6A**). We further injected small amounts of IL-6 and IL-19 (10 ng/mouse) intradermally into *Socs3* deficient skin, and pathological signatures were assessed by histological analysis with K5 and MPO staining 14 days after the injection (**Fig.** **6B**). This amount of IL-6 and IL-19 is not sufficient to induce skin diseases in wild type mice. However, in *Socs3* cKO mice, both IL-6 and IL-19 independently caused epidermal hyperplasia and massive neutrophil migration, while control treatment (PBS) showed no pathological changes (**Fig.** **6B**); an indication that IL-19 expression in the keratin layer led to the skin inflammation through the attraction of neutrophils.

We further investigated whether IL-20R related cytokines are required for the IL-6 induced epidermal hyperplasia. We first tested the inhibitory properties of two independent reagents, IL-20Rβ-Fc fusion protein and anti-IL-20Rα antibody. The inhibitory activity was assessed by IL-6 production from IL-19 activated keratinocytes, and the inhibition was observed with the IL-20Rβ-Fc fusion protein (**Fig.** **S3**). Next, *Socs3* deficient mice were injected intradermally with IL-6 (20 ng/mouse) along with either control Ig or the IL-20Rβ-Fc fusion protein, and pathological signatures were assessed as epidermal hyperplasia at fourteen days after treatments. Treatment with the IL-20Rβ-Fc fusion protein completely abrogated the IL-6 induced epidermal hyperplasia (**Fig.** **6C** **)**. The same inhibition also observed in the mice treated with anti-IL-20 antibody (**Fig.** **S4**). These results demonstrated that IL-6-induced pathogenesis in the skin disease occurs though the induction of expression of IL-20R related cytokines.

### Physical stimuli induced expression of IL-20R related cytokines and hyperplasia in Socs3 cKO mice

**Figure 7:**
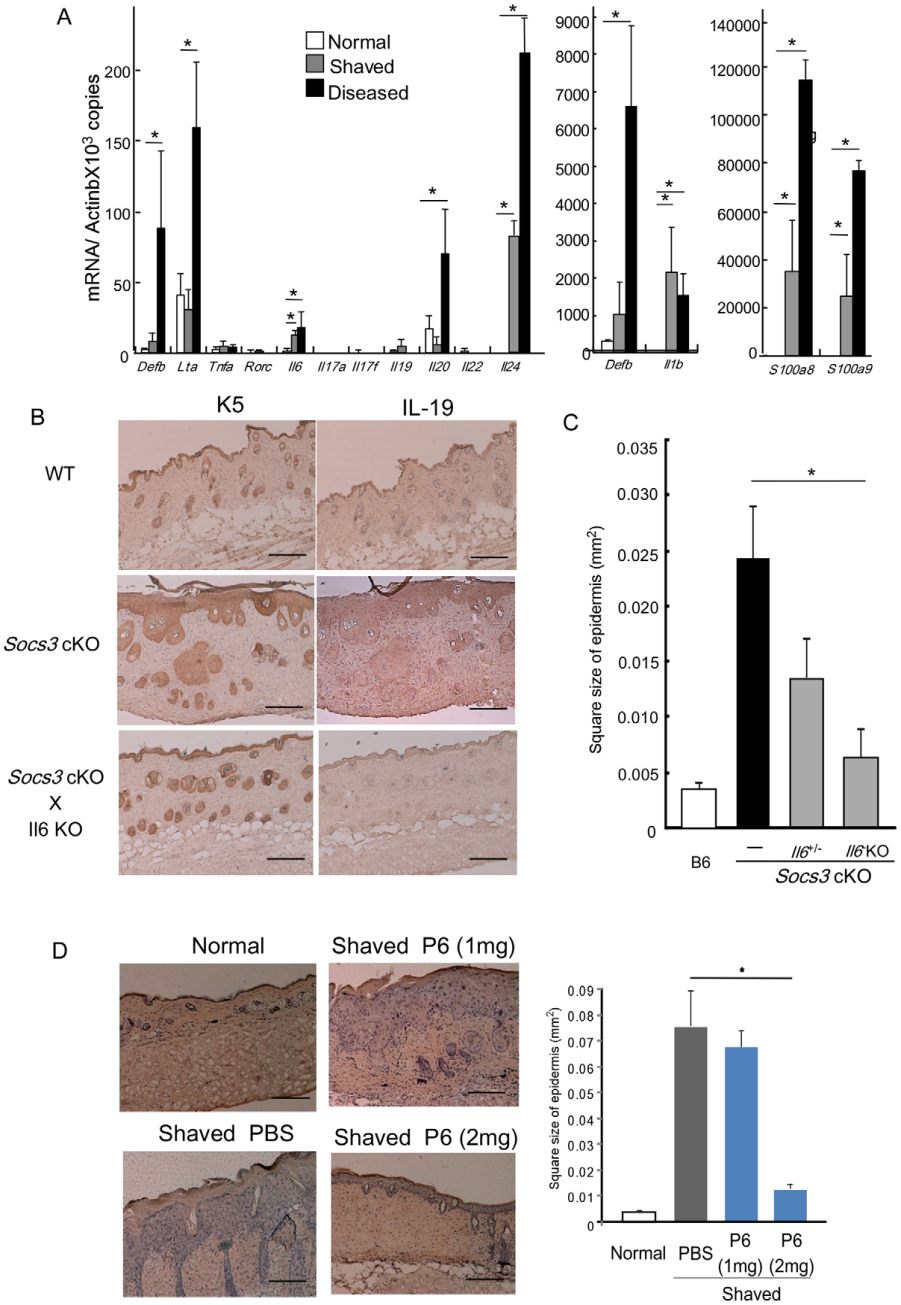
Physical stimulation initiates epidermal hyperplasia. A) mRNA was prepared from the skin of healthy K5-Cre (Normal), the shaved *Socs3* cKO mice (Shaved) and the diseased *Socs3* cKO mice (Diseased), and analyzed for the expression of the indicated genes by SYBR green real-time qPCR analysis. B) Immunohistochemical staining of keratinocytes (K5, left panels) and IL-19 positive cells (IL-19, left panels) in epidermis and dermis of K5-Cre (WT), *Socs3* cKO mice and *Socs3* cKO mice crossed with *Il6* KO mice (*Socs3* cKO X *Il6* KO) (x40). Scale bar in each section indicates 750 υm. C) The role of IL-6 on the development of epidermal hyperplasia in *Socs3* cKO mice. B6, *Socs3* cKO mice, *Socs3* cKO mice crossed with *Il6* ^+/−^ or *Il6* KO mice were studied. The square area of epidermis (0.25 mm^2^) in the section was measured at day 5 after shaving and is indicated on the Y-axis. Data are mean of the square size and error bars indicate SEM (n = 3). D) Effect of PLGA-P6 on physical stimulation-induced epidermal hyperplasia in *Socs3* cKO mice. Left; The dorsal skin area of *Socs3* cKO was shaved with depilatory cream and PLGA-P6 (1 mg or 2 mg) or PBS was injected intradermally into the shaved area. The shaved area (Shaved) was then compared to the non-treated area (Normal). At day 5 after shaving, skin sections were examined by H&E staining to assess the appearance of hyperplasia. The images are representative of three independent experiments (x40). Scale bar in each section indicates 750 υm. Right; the square size of the epidermis (0.25 mm^2^) in the sections shown in the left image was measured at day 5 after shaving and is indicated on the Y-axis. Data are mean of the square size and error bars indicate SEM (n = 3).

Skin lesions of *Socs3* cKO mice were consistently observed on the head and face, where mice were able to scratch by themselves, suggesting that physical stimulation by scratching might be a key event responsible for the disrupted skin barrier. To examine this possibility, artificial stimulation was provided by shaving hair on the dorsal skin where mice are unable to directly scratch. Five days after shaving, *Socs3* cKO mice, but not control mice, exhibited scaly skin and scabs at the shaved site, and the level of IL-19, IL-20 and IL-24 expression was comparably increased (**Fig.** **7A**). *Socs3* KO skin showed signs of epidermal hyperplasia on day 4 (**Fig.** **7B&C**), and this symptom completely resolved by day 14. Therefore, in the absence of SOCS3, shaving is sufficient for the induction of hyperplasia. The hyperplasia was only partially resolved in *Socs3* KO mice crossed with *Il6* ^+/−^ mice, but was completely resolved in *Socs3* KO mice crossed with *Il6* KO mice. *Il6* KO skin exhibited very weak expression of IL-19 in the shaved area (**Fig.** **7B**). These results clearly demonstrated that IL-6 is a critical cytokine in the initiation of the epidermal hyperplasia induced by physical stimulation.

The shaving-induced epidermal thickening was inhibited by Tetracyclic Pyridone 6 (P6), a pan-JAK inhibitor that has a higher sensitivity in the JAK1-STAT3 activation pathway [23]. A low dose of P6 (1 mg) showed no effect, while high dose (2 mg) treatment almost totally inhibited the shaving-induced epidermal thickening (**Fig.** **7D**). These results indicate that the epidermal hyperplasia induced by physical stimulation is exacerbated through hyperactivation of the JAK-STAT pathway.

## Discussion

Recently, accumulating evidence has pointed to a crucial role for cytokines in chronic skin inflammation. The present study proposed that negative regulator for cytokine signaling SOCS3 plays a crucial role to maintain the keratinocyte homeostasis, and showed that defective SOCS3 expression causes inflammatory skin disease. We also found that the disruption of SOCS3 leads to excess activation of the IL-6-STAT3-IL-20R related cytokine signaling pathway. It is suggested that the balance among IL-6, STAT3 and SOCS3 controls normal skin homeostasis and maintains normal keratinocyte growth and proliferation. Imbalance of this skin homeostasis caused increased expression of IL-20R-related cytokines, eventually leading to the psoriatic inflammation in skin. Keratinocyte-specific Socs3 deficient mice provided us with a quite useful window on the disease process explained by the combined initiation of STAT3 activation and the IL-20R-related cytokine mediated psoriatic skin inflammation, and the understanding of the entire disease process.

Many skin diseases such as seborrheic dermatitis, atopic dermatitis and psoriasis involve inflammation, and in these skin disorders, T cells or T cell-derived cytokines are thought to associate with disease development and aggravation. However, our observations revealed that the dysregulation of cytokine signaling only in the keratinocytes is enough to induce severe inflammation. In *Socs3* cKO mice, the serum IgE level is up-regulated but this seems not to play a critical role in the disease development, suggesting that the up-regulation of IgE may be a secondary event in this skin disease.

In our study, IL-6 seems to be a trigger for the spiral of inflammation in *Socs3* KO skin. Regarding the source of IL-6 in the skin, neuropeptides are possible triggers. Several substances, including Substance P (SP) and vasoactive intestinal peptide (VIP) which is released from dermal nerve endings, are known to stimulate keratinocytes to produce cytokines including IL-6 [24]. These neuropeptides like VIP can be induced under various stressful conditions including mental stress, alcohol consumption, and smoking. Bacterial infection could be another case for IL-6 induction, because we found plaque of streptococcus in the inflamed skin area of some, but not all, *Socs3* cKO mice (data not shown). Bacterial infections cause an acute form of inflammatory response leading to elevation of IL-6 production in the skin.

In the diseased *Socs3* cKO mouse skin, STAT3 is highly activated but other STAT family members are not (data not shown), indicating that SOCS3 in keratinocytes specifically regulates STAT3 activity. Furthermore, improvement of the skin disease by treatment with the P6 pan-JAK inhibitor confirmed that key cytokine signaling here utilizes the JAK/STAT pathway. SOCS3 specifically inhibits IL-6-induced STAT3 activation by binding to the STAT3 docking site (Tyr759) in one of the components of the IL-6 receptor, gp130 [25], [26]. This pivotal role for SOCS3 in skin inflammation is supported by recent reports showing that a specific microRNA, miR203, is highly expressed in human psoriatic skin and inhibits the expression of SOCS3 [27]. IL-20R-related cytokines, IL-19, IL-20 and IL-24, transmit their signal through IL-20Rα/IL-20Rβ receptor complex. These cytokines have been reported to play important roles in the skin, and are often thought to be pro-inflammatory cytokines [28]. Here we report that IL-6 induced higher levels of IL-19, IL-20 and IL-24 mRNA in SOCS3 deficient keratinocytes than in the WT keratinocytes, consistent with IL-6 mediated IL-20 induction in human keratinocytes [29]. Together, these findings indicate that in the healthy condition, SOCS3 is required for inhibition of the IL-6 induced increases in pro-inflammatory IL-20R-related cytokines. The up-regulation of these cytokines leads to neutrophil accumulation directly or indirectly through the induction of neutrophil chemoattractants, S100A8/S100A9.

In conclusion, we have demonstrated that skin homeostasis is maintained by a balance among IL-6, STAT3 and SOCS3 in keratinocytes. Furthermore, when this balance is broken and STAT3 activation is out of control, inflammatory skin disease is induced without any abnormalities in the immune cells. This SOCS3 mediated homeostatic function plays a key role in negatively regulating STAT3 activity. The relation among IL-6, STAT3, and SOCS3 provides a useful tool for understanding the mechanism of chronic skin inflammation in humans.

## Materials and Methods

### Reagents

Polylactide-glycoside (PLGA) nanospheres have been reported as useful pulmonary drug delivery carriers for improving the pharmacological effect of drugs [30]. The pan-JAK inhibitor Tetracyclic Pyridone 2-tert- butyl- 9-fliro- 3,6-dihydro- 7H-benz[h]-imidaz[4,5-f]isoquinoline-7-one (P6) [23] was packaged with PLGA nanospheres by Hosokawa Powder Technology Research Institute (Osaka). P6 was dissolved in PBS when applied onto the skin of mice. The dorsal skin area of *Socs3*cKO was shaved with depilatory cream, and P6 (1 mg or 2 mg) or PBS was injected intradermally into the shaved area. Five days after shaving, skin samples were prepared and frozen for sectioning and H&E staining.

### Mice

K5 Cre Tg [31], *Socs1* ^f/f^ [32], Socs3^f/f^ [33], Il23^−/−^ [34], Il4r^−/−^ [35], Rag1^−/−^ [36] and Il6^−/−^ [37] mice are described elsewhere. All mice used in this study except *Il4r* ^−/−^ were backcrossed into C57BL/6 mice, and *Il4r* ^−/−^ mice were maintained on a BALB/c background. C57BL/6 and BALB/c mice were purchased from CLEA Japan, Inc. (Tokyo, Japan). All mice were maintained in SPF conditions.

### Monitoring disease incidences

The clinical scoring was assessed with the incidence number of regions: 0, no lesion; 1, lesion in ear; 2, lesion in half of face; 3, lesion in whole face. The scoring was done until week 50.

### Antibodies and Cytokines

Immunohistochemistry was carried out with the following antibodies. Biotinylated rat anti-mouse CD4 (H129.19), biotinylated mouse anti-ClassII (I-A^b^) (KH74), rat-anti-IL-17A (TC11-18H10)-PE, mouse anti-pSTAT3 (4/P-STAT3)-Alexa 488 and rat anti-IFNγ (XMG1.2)-PE antibodies were purchased from BD Biosciences (San Diego, CA). Biotinylated rat anti-CD8 (53–6.7), biotinylated hamster anti-CD11c (N418), rat-anti-Langerin (eBioL31), biotinylated rat anti-FcεRIα (MAR-1) and rat anti-IL-20R2 (20RNTC) were purchased from e-Biosciences (San Diego, CA). Goat anti-IL-6 (AF-406-NA), rat anti-IL-19 (350105), recombinant murine IL-23R Fc chimera (1686-MR), rat anti-IL-24 (303308), rat anti-IL-20 (380605) antibodies and recombinant murine IL-20Rβ Fc chimera (4388-MR) were purchased from R&D Systems, Inc. (Minneapolis, MN). Rabbit anti-pSTAT1 (9171), anti-pSTAT5 (9351) and anti-pSTAT6 (9361) were purchased from Cell Signaling Technology (Massachusetts, MA). Rabbit anti-K5 (PRB-160P) was obtained from Covance Research Products Inc. (Denver, PA). Goat anti-PAR2 (sc-8205) was purchased from Santa Cruz Biotechnology, Inc. (Santa Cruz, CA). Rabbit MPO polyclonal antibody (PA1-28215) was obtained from Pierce Biotechnology (Rockford, IL). Rat anti-MCP-8 (TUG8) was purchased from BioLegend (San Diego, CA). Rabbit anti-IL-20Rα antibody (ab25922) was obtained from abcam (Cambridge, UK). Murine anti-rat and goat antibodies (Histo-fine MAX-PO), Histo-fine streptavidin, anti-rat IgG Alexa Fluor 488 and streptavidin Alexa Fluor 546 (Invitrogen), anti-human IgG HRP (Jackson immune research, Pennsylvania, PA) were used for 2^nd^ antibodies for Immunohistochemistry. Recombinant murine IL-6 and IL-19 were purchased from Peprotech Inc. and R&D Systems, Inc., respectively.

### In vitro keratinocyte culture

Primary keratinocytes were obtained from epidermis isolated from mouse ears. The epidermis was separated from the dermis following 1 hour incubation at 37°C in 0.25% trypsin/EDTA (Nacalai Tesque, Kyoto Japan) followed by filtration through 70 υm nylon mesh cell strainers (BD). Keratinocytes were suspended in the keratinocyte growth media (KGM-2, Lonza, Basel, Switzerland) containing 4 ng/ml mouse epidermal growth factor (EGF) and seeded at 5×10^5^ cells/ml. Before cytokine stimulation, keratinocytes were deprived of growth factors for 4 hours, then stimulated with recombinant IL-6 (10 ng/ml). After 10 or 60 min stimulation, cells were harvested and intracellular staining of pSTAT3 was performed. The flow cytometric analysis was done with a FACS Calibur (BD). For the detection of mRNA expression, after 24 hours stimulation, cells were harvested and RNA was extracted with RNAeasy mini kit (Qiagen). RT-PCR was done with SYBR green real-time quantitative PCR assay.

### Histology and immunohistochemistry

The frozen skin sections were fixed with acetone and then stained with H&E. After blocking the sections with 3% BSA/PBS for 30 min, they were treated with primary abs in 1% BSA/PBS and were incubated for 30 min at room temperature. When the secondly abs were labeled with HRP, sections were then visualized by using DAB for 5 to 15 minutes. After washing the sections with water for 10 mins, sections were treated with EtOH and xylene and observed under light microscopy. When the secondly antibodies were fluorescently labeled, the sections were washed with PBS(−)−0.05% Tween20 after secondly antibody treatment. After washing the sections with water for 10 mins they were mounted with VECTA SHIELD/Prolong Gold reagent and imaged using an LSM 510 confocal microscopy system (Carl Zeiss, Germany). For the quantification of mast cells, basophils and neutrophils, toluidine blue, MCP-8 and MPO were used as cell markers respectively. We randomly picked four fields on the sections from each mouse for analysis.

### Gene expression analysis

For gene expression analysis, total RNA was isolated using RNA STAT-60 (Tel-Test, Friendswood, TX, USA). Total RNA (5 υg) was subjected to treatment with DNase (Roche). DNase-treated total RNA was reverse-transcribed using Superscript II (Gibco/BRL). Primers were designed using Primer Express (PE Biosystems), or obtained commercially from Applied Biosystems. Real-time quantitative PCR on 10 ng of cDNA from each sample was performed using either of two methods. In the first method, two gene-specific unlabelled primers were utilized at 400 nM in a Perkin Elmer SYBR green real-time quantitative PCR assay utilizing an ABI 5700 instrument. In the second method, two unlabelled primers at 900 nM each were used with 250 nM of FAM-labeled probe (Applied Biosystems) in a TAQMAN^TM^ real-time quantitative PCR reaction on an ABI 7700 sequence detection system. The absence of genomic DNA contamination was confirmed using primers that recognize the genomic region of the CD4 promoter – samples with detectable DNA contamination by real-time PCR were excluded from the study. Ubiquitin levels were measured in a separate reaction and used to normalize the data by the Δ – Δ Ct method, using the mean cycle threshold (Ct) value for ubiquitin and the gene of interests for each sample; the equation 1.8 e (Ct ubiquitin – Ct gene of interest) x 10^4^ was used to obtain the normalized values.

### Air pouch

C57BL/6 mice (8–9 wks) were injected with 5 ml of sterile air into the subcutaneous tissue of the back, followed by a second injection of 3 ml of sterile air into the pouch 3 days later. IL-19 (1 mg) in 1 ml of sterile PBS, or sterile PBS as a control were injected into the pouch 7 days after the first injection of air. After 5 hrs, mice were killed and pouch fluids were harvested by injecting 0.5 ml of PBS. After centrifugation, the supernatant were analyzed for cytokines/chemokines concentrations with Bioplex Cytokine assay kit (Bio-Rad Laboratories, Hercules, CA, USA) according to the manufacturer's protocol.

## Supporting Information

Figure S1 **Skin sections from the diseased Socs3 cKO mice were stained with anti-pSTAT1, anti-pSTAT5, or anti-pSTAT6, and the sections were further probed with HRP labeled secondary antibody. Control indicates secondary antibody alone.**
(TIF)

Figure S2 **Expression of IL-17A and IL-23 protein in frozen sections of K5-Cre control (WT) and the diseased skin from Socs3 cKO (cKO) mice was analyzed by immunohistochemical staining. The images are representative of five independent experiments (x40).**
(TIF)

Figure S3 **Isolated keratinocytes were cultured with IL-19 in the presence of IL-20Rβ-Fc fusion protein or anti-IL-20Rα antibody. After 6** **hrs, IL-6 production in culture supernatant was measured by ELISA. Data are mean and SEM of three independent cultures.**
(TIF)

Figure S4 **Socs3 deficient mice were injected intradermally with IL-6 (10** **ng/mouse) with either control Ig (isotype) or anti-IL-20 antibody (100** **υg).** After two weeks, skin sections were stained with H&E and epidermal thickness was measured at the injection site (x200). Scale bar in each section indicates 150 υm. Bar graph (right panel) indicates the mean and SEM (n = 3) of epidermal thickness (υm).
(TIF)
