# Supplementary figures and images for: Dysregulation of Suppressor of Cytokine Signaling 3 in Keratinocytes Causes Skin Inflammation Mediated by Interleukin-20 Receptor-Related Cytokines
Source: PLoS One. 2012 Jul 5;7(7):e40343. doi: 10.1371/journal.pone.0040343 (PMC3390387; doi:10.1371/journal.pone.0040343)

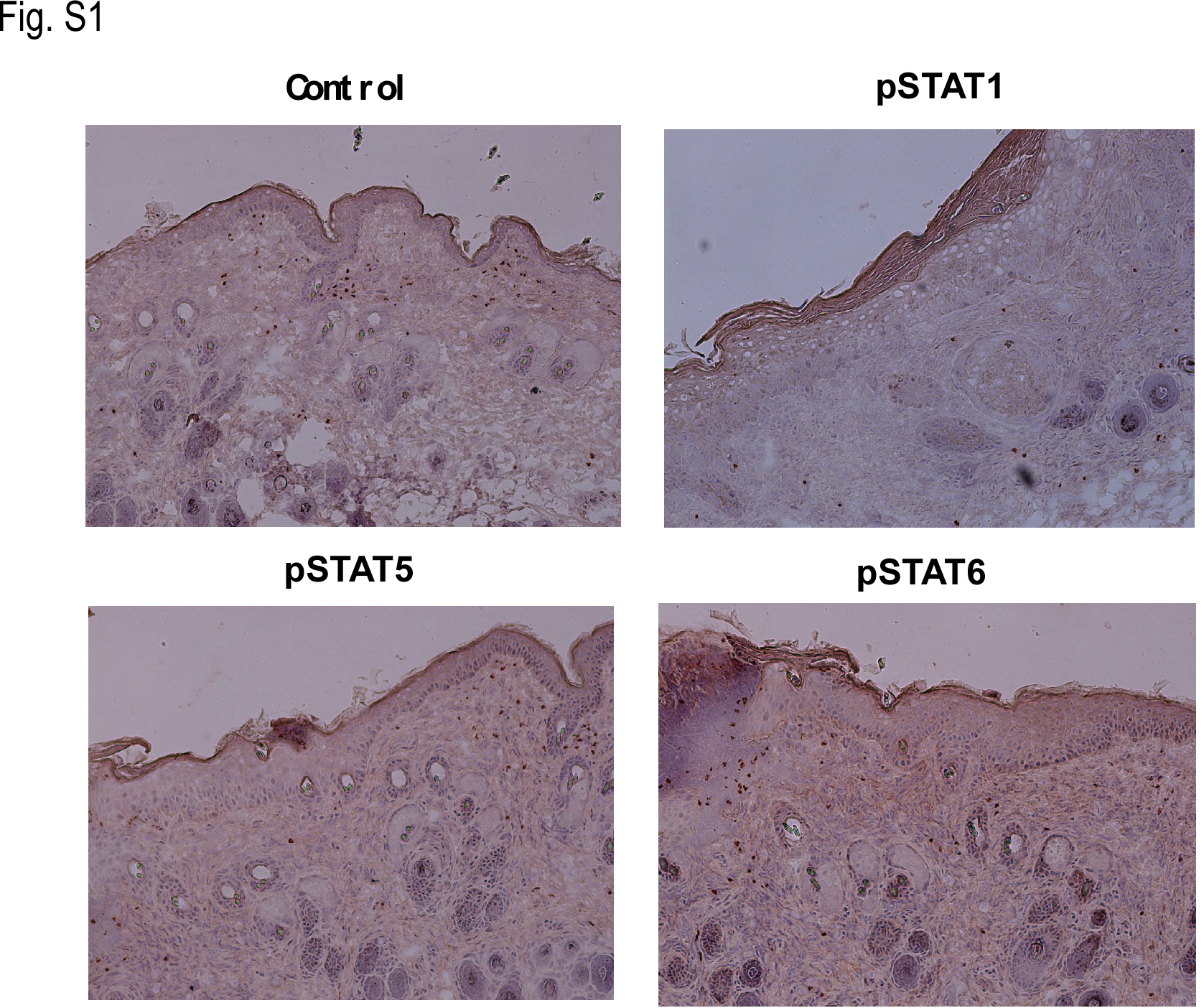

Supplement: Figure S1 — Skin sections from the diseased Socs3 cKO mice were stained with anti-pSTAT1, anti-pSTAT5, or anti-pSTAT6, and the sections were further probed with HRP labeled secondary antibody. Control indicates secondary antibody alone. (TIF) [file pone.0040343.s001.tif]

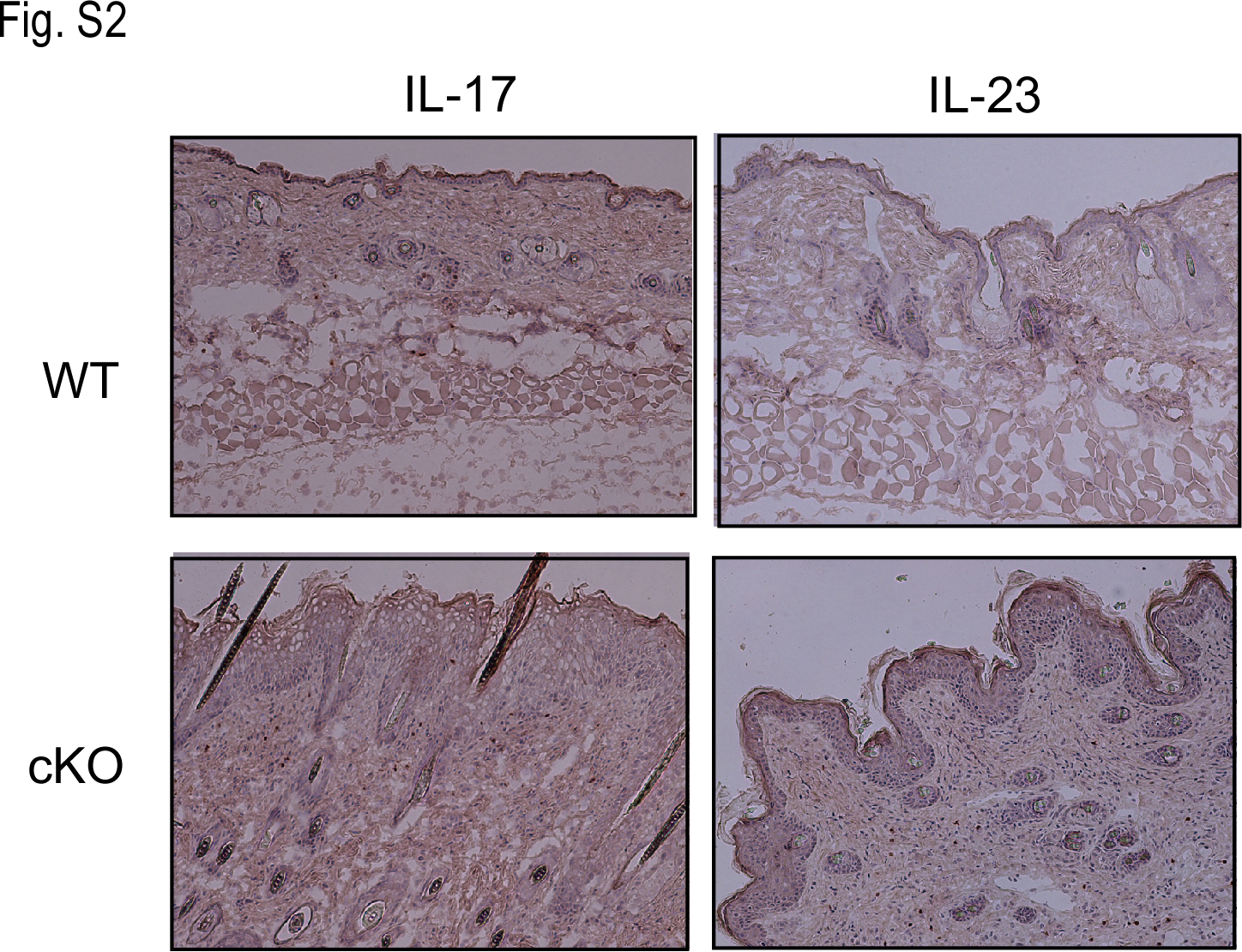

Supplement: Figure S2 — Expression of IL-17A and IL-23 protein in frozen sections of K5-Cre control (WT) and the diseased skin from Socs3 cKO (cKO) mice was analyzed by immunohistochemical staining. The images are representative of five independent experiments (x40). (TIF) [file pone.0040343.s002.tif]

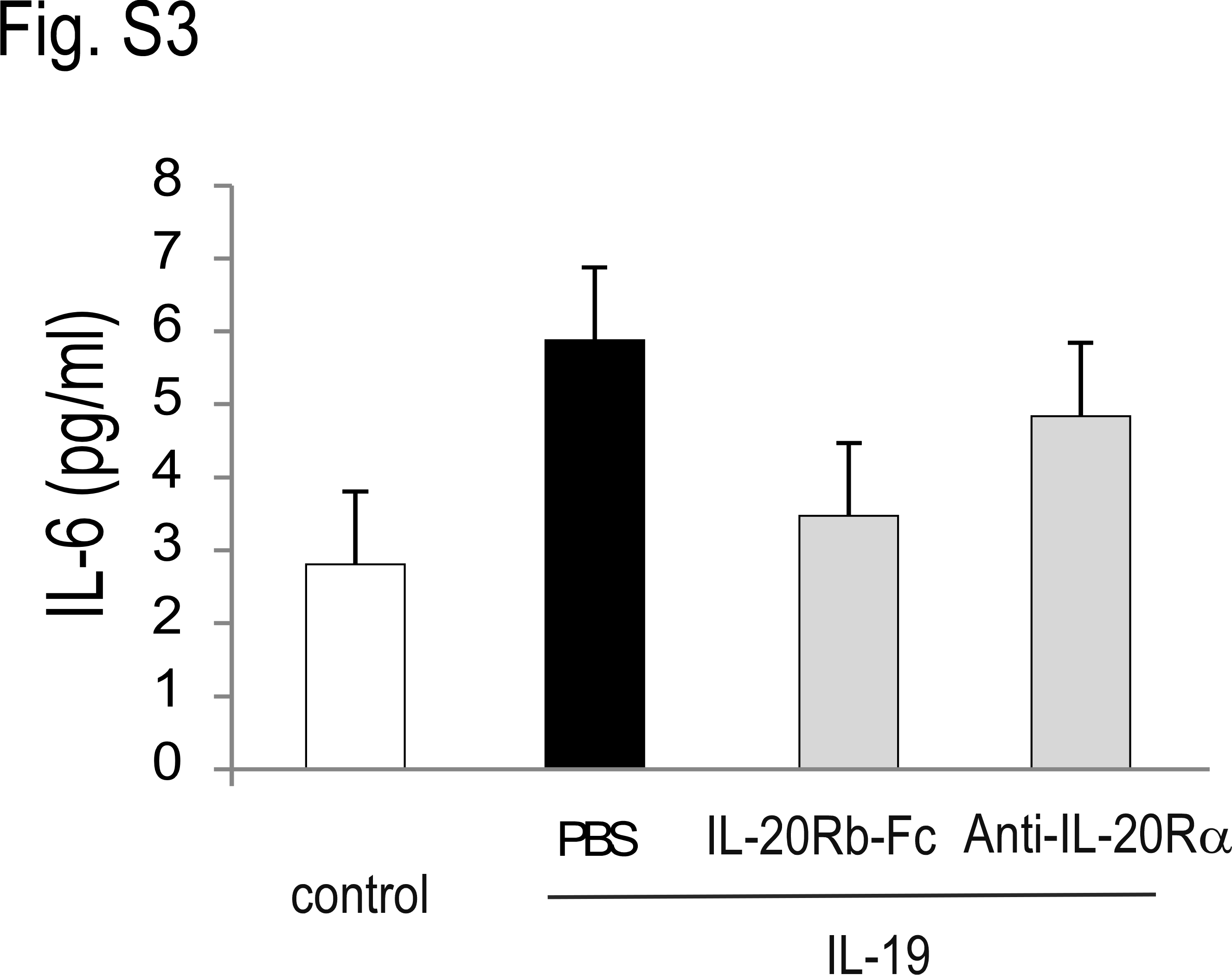

Supplement: Figure S3 — Isolated keratinocytes were cultured with IL-19 in the presence of IL-20Rβ-Fc fusion protein or anti-IL-20Rα antibody. After 6 hrs, IL-6 production in culture supernatant was measured by ELISA. Data are mean and SEM of three independent cultures. (TIF) [file pone.0040343.s003.tif]

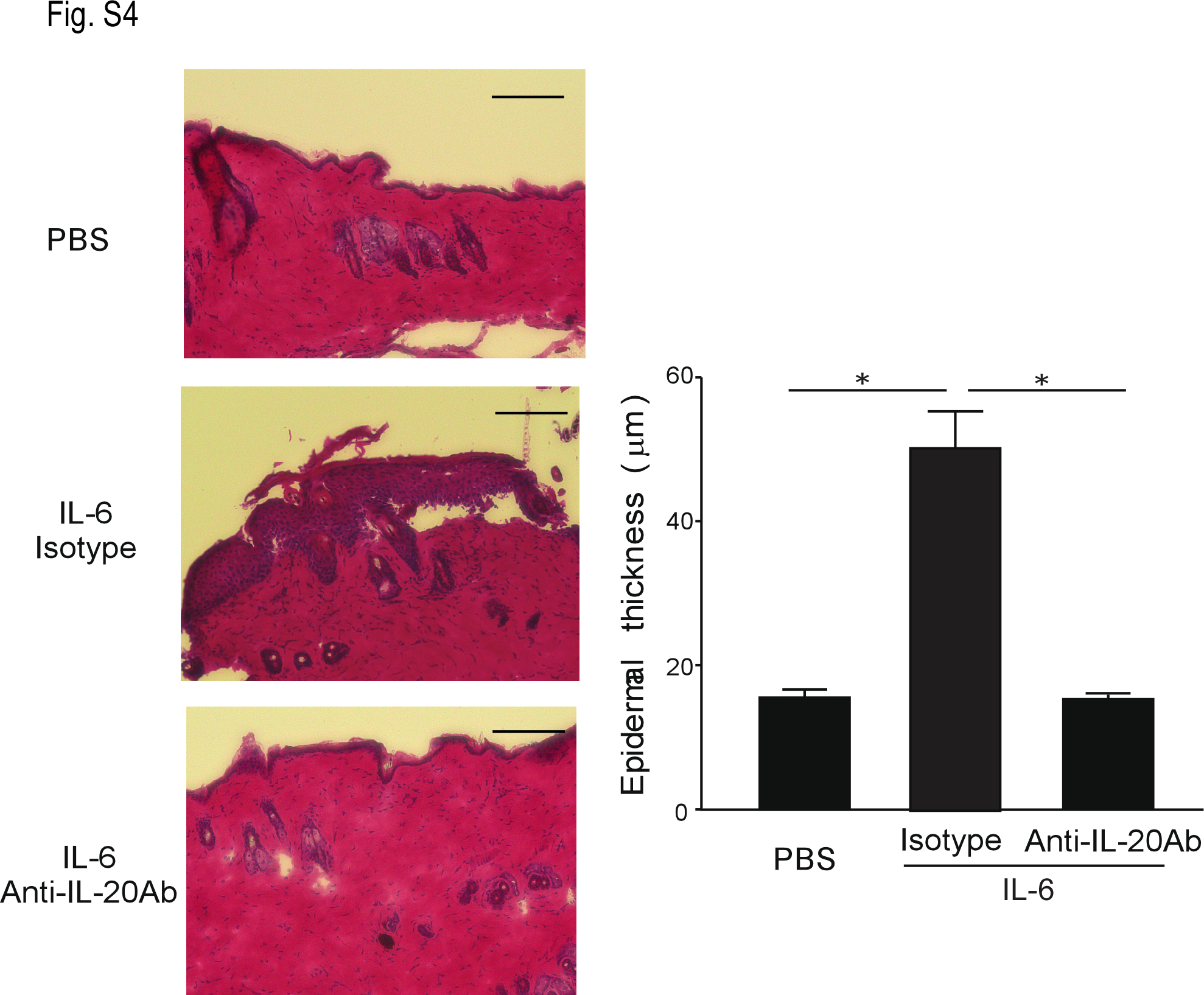

Supplement: Figure S4 — Socs3 deficient mice were injected intradermally with IL-6 (10 ng/mouse) with either control Ig (isotype) or anti-IL-20 antibody (100 υg). After two weeks, skin sections were stained with H&E and epidermal thickness was measured at the injection site (x200). Scale bar in each section indicates 150 υm. Bar graph (right panel) indicates the mean and SEM (n = 3) of epidermal thickness (υm). (TIF) [file pone.0040343.s004.tif]
